# The Fate of Allogeneic Pancreatic Islets following Intraportal Transplantation: Challenges and Solutions

**DOI:** 10.1155/2018/2424586

**Published:** 2018-09-23

**Authors:** Xinyu Li, Qiang Meng, Lei Zhang

**Affiliations:** Department of General Surgery, The 2nd Affiliated Hospital of Harbin Medical University, 246 Xuefu Road, Harbin, 150086 Heilongjiang Province, China

## Abstract

Pancreatic islet transplantation as a therapeutic option for type 1 diabetes mellitus is gaining widespread attention because this approach can restore physiological insulin secretion, minimize the risk of hypoglycemic unawareness, and reduce the risk of death due to severe hypoglycemia. However, there are many obstacles contributing to the early mass loss of the islets and progressive islet loss in the late stages of clinical islet transplantation, including hypoxia injury, instant blood-mediated inflammatory reactions, inflammatory cytokines, immune rejection, metabolic exhaustion, and immunosuppression-related toxicity that is detrimental to the islet allograft. Here, we discuss the fate of intrahepatic islets infused through the portal vein and propose potential interventions to promote islet allograft survival and improve long-term graft function.

## 1. Introduction

Insulin deficiency caused by autoimmune injury of islet *β* cells is the primary cause of type 1 diabetes mellitus (T1DM). Islet replacement therapy (insulin-secreting pancreas transplantation or islet transplantation) enables the physiological regulation of blood glucose and precise maintenance of glycemia, which is not attainable by other modern interventions, including insulin pumps and/or continuous blood glucose monitoring therapies. Compared with pancreas transplantation, islet transplantation exhibits great promise due to its safe and minimally invasive process and is a sought-after option for the treatment of T1DM.

**Figure 1:**
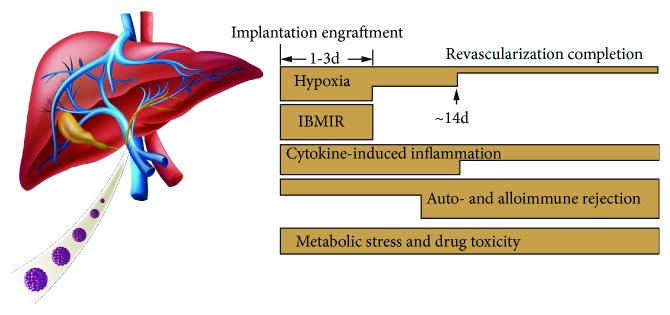
Time frame of detrimental factors leading to early injury and late loss of function after islet transplantation is shown. Massive tissue loss due to IBMIR early during transplantation reduces successful engraftment. Islets endure a severely hypoxic environment in the first several days and rely only on passive oxygen diffusion for survival.

Over the past 20 years, significant progress has been made in the management of islet cells and the outcome of clinical islet transplantation. In some leading islet transplant centers, it is possible to achieve 5-year insulin independence rates of 50–70%, on a par with whole-pancreas transplantation in T1DM patients [1–5]. However, many challenges remain in clinical islet transplantation. In this review, we focused on the fate of the islets infused through the portal vein, which are subjected to multiple insults, including anoxia/ischemia-reperfusion injury, instant blood-mediated inflammatory reaction (IBMIR), potent autoimmune and alloimmune rejections, metabolic exhaustion, and immunosuppression-related toxicity (Figure 1). We also propose protective strategies to circumvent these adverse events to alleviate the loss of islets and improve the long-term outcomes of transplantation.

## 2. Hypoxia

Due to their high oxygen dependence and lack of ability to scavenge free radicals, islets are particularly vulnerable to hypoxia [6]. Revascularization is imperative for long-term survival of dispersed islets in the hepatic vascular network. This process usually takes 10–24 days [7], and vascular remodeling can take up to three months [8].

Before the vessels grow into the islets, the survival of the islets primarily depends on the passive diffusion of nutrients and oxygen; thus, the larger the islet diameter, the more susceptible the central cells are to hypoxia.

Cultured islets in vitro often appear darkened in the central region when observed under an inverted microscope. This phenomenon is defined as central cell necrosis, which is often associated with reduced islet function [9]. Smaller islets are therefore favorable for transplantation and might lead to better graft survival [10]. Even after vascularization is complete, the vascular density, oxygen tension, and blood perfusion of the engrafted islets remain in an anoxic state compared with native islets [11–13]. Prolonged hypoxia during islet transplantation initiates a cascade of biochemical reactions causing the production of reactive oxygen species and the induction of apoptosis and necrosis via intracellular pathways [14]. Additionally, hypoxia and reperfusion of oxygen induce the release of multiple proinflammatory mediators from islets, such as high mobility group box-1, regulated upon activation of normal T cell expressed and secreted, tumor necrosis factor-*α* (TNF-*α*), interleukin-1*β*, interferon gamma, monocyte chemoattractant protein-1, tissue factor (TF), and macrophage inflammatory protein 1*α* [15, 16], which amplifies inflammation that, in turn, impairs islet survival.

### 2.1. Strategies to Overcome Islet Hypoxia

A variety of methods have been proposed to promote the survival and improve the function of islets in an anoxic environment. These methods can be divided into two categories: (1) protection of islets from hypoxic injury and (2) increased oxygen supply to islets to prevent hypoxia.

Some studies have shown that gene modification can endow islets with resistance to hypoxia by inhibiting apoptotic triggers. For example, heme oxygenase-1 [17, 18], A20 [19–21], B cell lymphoma 2, and X-linked inhibitor of apoptosis [22] have been identified to prevent/alleviate islet apoptosis and improve islet survival under in vitro and in vivo experimental conditions but remain to be validated in clinical studies.

Detrimental oxidative products released by hypoxia, such as inducible nitric oxide synthase and reactive oxygen species, promote the expression of proapoptotic genes (i.e., Fas and Bax), resulting in rapid apoptosis or necrosis of *β* cells [23]. Treatment of islet grafts with potent antioxidants can mitigate oxidative stress. Enicostemma littorale methanol extract can protect islets from oxidative stress-induced cell death in vitro [24]. A redox-active metalloporphyrin, BMX-001, was shown to enhance islet viability, reduce apoptosis in vitro, and improve marginal islet mass engraftment in diabetic mouse models [25]. Controlling oxidative stress may improve islet survival.

The second category includes accelerating the islet vascularization process posttransplantation and increasing oxygen content at the transplant site. High levels of vascular endothelial growth factor (VEGF) expression in islets and vascular endothelial cells contribute to neovascularization [8, 26]. The addition of VEGF to islet grafts has both positive and negative effects, as VEGF also expedites and amplifies inflammation, which is harmful to the survival of the islets. Lee et al. reported that VEGF-transfected islets could enhance islet vascularization and graft function in STZ-induced diabetic mice [27]. Hepatocyte growth factor, fibroblast growth factor, epidermal growth factor, and biomaterials can also favor isolated islet angiogenesis [28]. Uematsu et al. used a novel scaffold, recombinant peptide to optimize prevascularization procedures to augment subcutaneous islet function in mice [29]. A 3D-printed vascularized device has been invented and has enabled the long-term survival of human islets subcutaneously in immune-deficient mice [10]. Oxygen feeding to the transplanted islet is an intuitive means to overcome hypoxia. In this case, the islets are usually stored in a bioartificial pancreas device and the de novo generated oxygen is produced by electrochemistry [30]/photosynthesis [31] or exogenous oxygen and is delivered into the device for islet use. Hyperbaric oxygen therapy has been used for islet transplantation in mice [32–35] and for autologous stem cell infusion in patients with type 2 diabetes mellitus [36], providing therapeutic potential for human islet transplantation. Most of the abovementioned techniques are still at different stages of preclinical trials.

## 3. IBMIR

Thus far, intraportal islet infusion remains the optimal approach for clinical transplantation. A large number of islets are innately destroyed by an event termed IBMIR on contact with the recipient's blood. It is estimated that approximately 60–70% of islets are lost prior to hepatic engraftment, which is the main cause of “primary nonfunction” and the need for 2–3 pancreas donors to achieve euglycemia in the initial period following transplantation [37, 38].

The thrombotic/inflammatory reaction is a cascade reaction that start with the coagulation and complement system activation, activated platelet adhesion to the islet surface, and abundant leukocyte infiltration into the islets with structure integrity disruption [39, 40].

**Figure 2:**
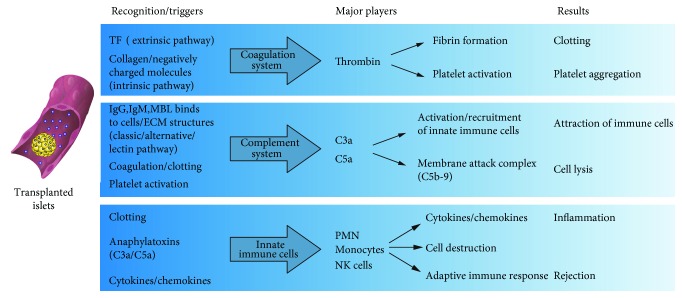
Coagulation, complement, and immune cells interact to orchestrate IBMIR, the primary cause of early massive loss of transplanted islets. Figure modified from Digital Comprehensive Summaries of Uppsala Dissertations from the Faculty of Medicine 1030.

The functional impairment and death of pancreatic islets by IBMIR is usually ascribed to multiple pathological effects. First, the infiltrating cells (neutrophils and macrophages) are directly cytotoxic to the islet cells [41, 42]. Second, inflammatory cytokines lead to apoptosis and necrosis of the islet cells [43]. Finally, IBMIR potentiates and amplifies the subsequent cell-mediated immune response (Figure 2) [42, 44, 45].

### 3.1. Strategies for Alleviating IBMIR

According to the reaction characteristics, coagulation, complement activation, and inflammatory processes can be taken as individual and/or combined intervention targets. Potential approaches to relieve stress and protect islets can be achieved through pretreatment of islets in vitro and systematically administered with anticoagulants, complement inhibitors, anti-inflammatory reagents, or islet surface engineering (Table 1). We will discuss these measures briefly below.

Both pretreatment and gene modification are capable of weakening the procoagulant and proinflammatory status of pancreatic islets to minimize the deleterious outcomes of IBMIR. TF serves as the main trigger of IBMIR and can inhibit blood coagulation by reducing the expression level of TF. Islets pretreated with nicotinamide in vitro can downregulate TF, monocyte chemoattractant protein-1, and other inflammatory cytokines dramatically [46]. Due to the extensive nature of events associated with IBMIR, multiple genetic modifications may be required to provide adequate graft protection.

The main purpose of gene manipulation in human islets in vitro is to introduce antiapoptotic genes and antioxidant genes to enhance the resistance of transplanted islets to inflammation-induced injury, which constitutes a major component of IBMIR. For example, B cell lymphoma 2-transfected human islets were obviously protected from cytokine-induced dysfunction in vitro [47].

Multiple genes are modified simultaneously in an individual islet to resist the harmful effects of the coagulation and complement system, and the use of a transgenic donor is the best solution. In this respect, pig islet xenotransplantation presents remarkable advantages and significant progress has been made. Pigs without TF and pigs expressing a human “antithrombotic” or “anticoagulant” gene, such as thrombomodulin, TF pathway inhibitor, or CD39, are available to minimize IBMIR and coagulation dysfunction [48].

Systematic application of heparin or low-molecular-weight dextran sulfate and soluble complement receptor-1 can improve islet survival by downregulating allogeneic IBMIR in experimental settings but remains to be validated in clinical studies [38, 49–51]. Controlling the inflammatory response can also alleviate IBMIR. Gala-Lopez et al. have shown that double blockade of interleukin-1*β* and TNF-*α* can significantly improve the efficiency of clinical islet transplantation, particularly in single-donor islet transplantation [52]. Recent studies demonstrated that both the CXC chemokine receptors 1/2 inhibitor reparixin [53] and serine protease inhibitor *α*1-antitrypsin [54] improved intrahepatic islet transplantation outcomes in mice and human trials, further confirming the efficacy of anti-inflammatory strategies, e.g., peritransplantation, that have been an essential component of current clinical islet transplantation. Surface engineered islets have been proven to ameliorate islet survival after islet transplantation by portal vein. For instance, heparin-coated islets attenuate the IBMIR and lead to more islets survived both in vitro loop model and in vivo pig model mimicking allogeneic intraportal islet transplantation [55]. Urokinase-, thrombomodulin-, and soluble complement receptor 1-coated islets can attenuate coagulation and complement activation when exposed to blood [56–58]. Human aortic endothelial cells conjugated to the islet surface significantly reduce all of the deleterious reactions of the IBMIR [59]. Although these techniques exhibit the greatest potential for islet preservation under experimental conditions, further investigation and evaluation are needed in clinical therapy.

## 4. Autoimmunity Recurrence and Alloimmunity

The islet allografts implanted in patients with T1DM are subjected to at least two separate categories of immune responses: (1) autoimmune T cell response and (2) conventional host antigraft immune response.

### 4.1. Autoimmunity

The pathogenesis of T1DM similarly affects the newly implanted islet grafts. The silent original autoreactive T cells with immune memory are reawakened by identical antigen reexposure after islet transplantation and trigger an attack on the graft, which could be supported by the fact that syngeneic *β* cells were damaged in autoimmune diabetic recipients [60]. The precise role of autoantibodies against *β* cell autoantigens, such as insulin-specific autoantibodies, glutamic acid decarboxylase, insulinoma antigen, and zinc transporter-8, in the pathogenesis of type 1 diabetes is unclear but of great significance to the prediction and diagnosis of T1DM [61]. People with 2 or more autoantibodies are more likely to develop T1DM than those with a single autoantibody [62]. However, the predictive ability of these antibodies in patients with islet transplantation remains controversial. Early studies of the immune response after islet transplantation have shown no correlation between preexisting autoreactive antibodies and graft dysfunction [63, 64], but later evidence demonstrated that patients with preformed autoantibodies have earlier islet graft loss than recipients without antibodies [65]. In contrast, autoreactive T cells (including CD4 and CD8 T cells) represent crucial players in the destruction of *β* cells and are the active intervention targets for therapy.

### 4.2. Alloimmunity

Alloimmunity is another major cause of transplanted islet destruction. Genetic diversity between the donor and the recipient determines the occurrence of immune response, which focuses primarily on human leukocyte antigen molecules in humans and major histocompatibility complex (MHC) in mice. T cell-mediated rejection constitutes the most important component in islet allotransplantation. The difference in human leukocyte antigen-1 antigens is the target of recipient CD8 T cells, and the difference in MHC-2 antigens is the target of recipient CD4 T cells [66].

**Figure 3:**
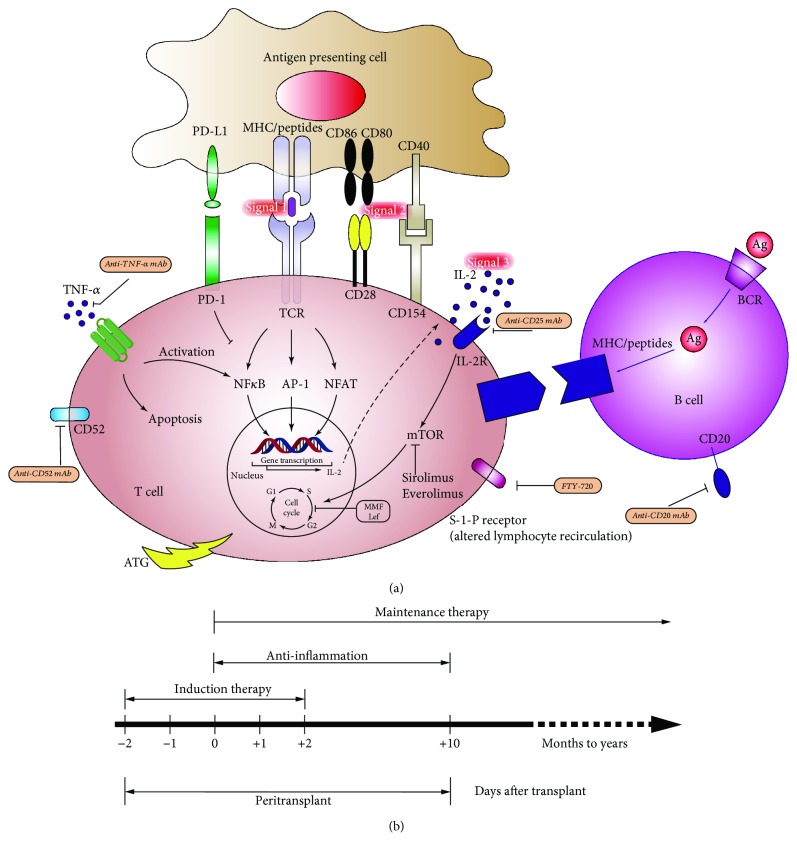
(a) The three-signal pattern of T lymphocytes activated in islet allotransplantation. Action targets and brief mechanisms are displayed. (b) Current immunosuppressive protocol commonly used in clinical islet transplantation associated with improved long-term islet allograft survival.

Whether the antigen is presented directly or indirectly, the activation of T cells is a critical step in rejection and requires the coordination of three signal systems (Figure 3(a)). That is, the first signal (peptide-MHC complex on the antigen-presenting cells), the second signal (costimulatory molecules such as B7-CD28 and CD40-CD154), and the third signal (cytokines) further amplify the proliferation of T cells [67]. The ultimate biological effect is the recruitment of immune cells to the grafts with function loss.

Patients undergoing allogeneic transplants can have humoral immunity, an antibody-mediated immune response, involved in the rejection process. Alloreactive antibodies are mainly directed towards MHC class I and MHC class II molecules. They include nonspecific antibodies called panel-reactive antibodies (PRAs) and donor-specific antibodies (DSAs). Both are recognized as predictive prognostic markers related to islet transplantation outcomes despite paradoxical and uncertain roles. Early studies have shown that pretransplant allogenic antibodies measured as PRA are a negative predictor of islet transplantation outcomes [68]. Pretransplantation PRA > 15% is associated with increased risk of C-peptide loss after islet transplantation [63, 69]. Islet transplantation outcomes in sensitized patients are often worse than those in nonsensitized patients [69]. Later, evidence did not support such a correlation between preformed PRA and islet function posttransplantation [70]. In contrast, there exists evidence that posttransplant-positive PRA levels and de novo DSA cannot predict islet transplantation outcomes [71].

### 4.3. Strategies for the Prevention of Auto- and Alloimmune Rejections

It is clear that both autoimmunity and alloimmunity contribute to the loss of islet function, which can occur separately or simultaneously in a single transplant process. Although remarkable progress in understanding the immune response mechanisms have occurred, it remains an important challenge to circumvent these response mechanisms in clinical settings. To date, suppression of T cells with globally immunosuppressive agents is the most widespread and practical approach (Table 2), as both production of antibodies and proliferation of B cells required the help of T cells. Inhibition of T cells, to some extent, can decrease the humoral immunity, although not completely. We will focus on cellular immunity in the following section.

Various immunosuppressive strategies have been examined in preclinical mouse models and are applied in the clinical setting; these therapies are divided into the induction and the maintenance of immunosuppression (Figure 3(b)). The principle of induced immunosuppression is to use preemptive means to maximize the consumption of T cells or inhibit the activation of T cells, including anti-CD3, antithymocyte globulin, or interleukin 2 receptor blockade prior to islet transplantation. Potent induction therapy with anti-CD3 Ab or T cell-depleting antibodies plus TNF-*α* inhibition is significantly associated with 5-year insulin independence of approximately 50% in islet transplant alone, comparable to outcomes in pancreas transplant alone, regardless of the choice of maintenance immunosuppression [1]. The maintenance of immunosuppression is more dependent on lifelong inhibition of T cell activation and proliferation, including tacrolimus, mTOR inhibitors, and mycophenolate mofetil [72], with the obvious drawbacks that most of these drugs exhibit liver and kidney toxicity and have direct toxicity to *β* cells. Of particular interest, costimulatory blockade represents a potential therapy that promotes immune tolerance, which has been demonstrated by several groups. For instance, short-term use of anti-CD154 monoclonal antibodies can lead to allograft tolerance when Balb/c islets are transplanted into STZ-treated C57BL/6 mice [73]. Moreover, anti-CD154 antibody plus other interventions, especially LFA-1 blockade, is highly effective [74]. Anti-CD154 antibody and LFA-1-induced tolerance in CD4 T-lymphocyte subsets can transfer to multiple islet transplant recipients [74]. Unfortunately, thrombotic events caused by anti-CD154 antibody hinder its clinical use. Newly developed anti-CD40 mAbs such as Chi220, ASKP40 (4D11), 3A8, and 2C10 may be equipotent but safe. Chi220 has been shown to extend allogenic islet survival time to more than 200 days in combination with belatacept (CTLA4-Ig) in MHC-mismatched rhesus macaques [75]. ASKP40 exhibits promise in retarding renal allograft rejection and antibody production in NHPs [76]. Badell et al. reported that 3A8 plus CTLA4Ig prevented DSA formation and potentially confers long-term islet allograft survival in alloislet nonhuman primate models [77]. NHP experiments with 2C10 also confirmed prolonged islet survival, with median graft survival time for animals receiving 2C10 of 280 days compared to 8 days for control animals [78]. The abovementioned results suggest the possibility that blockade of B7/CD28 signals and anti-CD40 mAb constitutes a promising immunosuppressive strategy to circumvent inadvertent thrombotic events caused by anti-CD154. Additional investigations are required to validate these findings in clinical human islet transplantation. Another option is to promote the expression of T cell inhibitory receptors on the islet. An example of such an approach is that surface engineered Ba/c islet grafts with the expression of FasL in conjunction with rapamycin treatment led to tolerance that was maintained by CD4(+) CD25(+) Foxp3(+) regulatory T cells (Tregs) in 100% of C57BL/6 recipients [79]. However, there exist contrary claims that the expression of the Fas ligand does not provide protection for grafts but instead exacerbates transplanted islet rejection [80, 81] and native islet autoimmune destruction [82]. In this way, its role in islet transplantation remains controversial [83]. Another example is a recently published report showing that enforced PDL-1 and CTLA-4 expression significantly prevented the development of autoimmune diabetes and delayed the rejection of the MHC-matched alloislet in STZ-induced diabetic mice >120 days (from Balb/c to DBA2), providing a potential strategy for immunosuppression-free islet transplantation [84].

Whether autoimmunity or alloimmunity represents a greater obstacle to islet transplantation success remains an elusive mystery. In clinical islet transplantation, the success of the Edmonton protocol was at least partly attributable to combined immunosuppressive regimens simultaneously blocking both auto- and alloimmune responses [85]. Other methods, including the use of biomaterials to encapsulate the islet, the infusion of donor bone marrow stem cells into recipients to produce hematopoietic chimeras [86], inducing Treg expansion in vitro and in vivo to alter the immune balance of Th1/Th2 [87], and combined infusion of donor mesenchymal stem cells [88] to enhance transplant efficacy may represent a path to induce immune tolerance or host nonresponse in autoimmune and allogeneic situations that, although experimental, is highly desirable.

## 5. Metabolic Exhaustion and Drug Toxicity

After the initial loss of a large number of islets, the residual islets may suffer progressive dysfunction. It is unclear whether the late progressive loss of function is due to immune factors or physical exhaustion.

However, for recipients undergoing autologous pancreatic islet transplantation following total resection of the pancreas, the fact that some recipients develop diabetes over time after achieving blood sugar homeostasis points to the possibility that the chronic islet dysfunction is likely due to physiological exhaustion rather than immune factors, especially in hosts harboring a marginal islet mass [89–91].

The metabolism of *β* cells is exceptionally high, constantly producing insulin-secreting granules. The fewer islets available for insulin production, the greater the metabolic pressure on each individual islet is, making the originally marginal islet grafts more dangerous. In addition, a major concern is the regeneration of transplanted islets. There is @little experimental data available to demonstrate how the islets change after transplantation. Whether *β* cells are regenerated from existing *β* cells or transdifferentiation by alpha cells or derived from islet intrinsic stem cells remains an open question. In any case, the isolated islets are deprived of nutrients and support from the surrounding cells and lose their primordial environment (the islets are scattered in the pancreas with surrounding exocrine tissue), which is detrimental to the regeneration and resistance of *β* cells and may explain why islet grafts are more vulnerable than the pancreas after transplantation.

Antirejection drugs can effectively suppress the immune response to the alloantigen but also increase the risk of life-threatening infections or malignant tumors. Furthermore, most immunosuppressive drugs, such as tacrolimus and mycophenolate mofetil, are detrimental to *β* cells, with direct toxicity, inhibition of insulin secretion, or the proliferation of *β* cells [92–95]. Thus, chronic use of these drugs will have negative impacts on grafts. Islet transplants can be deemed to be a “true cure” for diabetes only if no immunosuppressive drugs are applied.

With respect to reducing the metabolic load of islets, promoting the regeneration of *β* cells, reducing apoptosis, and improving the function of islets will be beneficial to the long-term survival of islets [96–99]. For drug-related toxicity, the development of new immunosuppressive drugs with less toxicity or the establishment of specific immune tolerance or immunomodulatory therapy may change the future treatment pattern in transplantation.

## 6. Concluding Remarks

Although insulin regimen, continuous insulin infusion, and strict blood glucose monitoring have made great progress in the treatment of diabetes mellitus, it remains difficult to achieve physiologically precise regulation of blood glucose. The replacement of permanently destroyed *β* cells with islet transplantation is the most logical treatment for T1DM and has proven to be very beneficial to patients. Islet transplantation can prevent severe hypoglycemia, improve haemoglobin A1C, prevent/reverse complications, and in many cases even achieve sustained periods of insulin independence. However, the prolonged use of immunosuppressive drugs increases the risk of infection, hepatorenal toxicity, and tumorigenesis, making the treatment less attractive and limiting it to patients with severe blood sugar instability in whom other therapies have failed. Successful islet allograft via the portal vein is hampered by limited islet survival after transplantation resulting from persistent anoxia, innate immunity attacks through IBMIR, recurrent autoimmune destruction or alloimmune rejection, sustained metabolic pressure, and drug toxicity. Optimizing islet revascularization with better control of angiogenesis, inhibiting inflammation, reducing oxidative stress, and promoting the regeneration of islet *β* cells can further improve the outcomes of islet survival. If these problems are properly addressed, hurdle limiting the wider use of islet transplantation in T1DM and partial type 2 diabetes mellitus will be the insufficiency of islet allograft donors.

The search for alternative sources of islets is therefore necessary. Efforts to improve islets from xenogeneic sources are ongoing, and remarkable progress has been made in the science and application of pluripotent stem cells, which are now in the early stages of clinical trials. New approaches such as the induction of specific immune tolerance and immune regulation with Treg infusion, mesenchymal stem cell cotransplantation, and innovative biological materials to protect islets from the immune system provide one feasible possibility for this therapy. The combination of genetically engineered porcine islets or pluripotent stem cells with immune isolation can solve both the organ shortage and immune rejection problems. Although the task remains challenging, success is possible. The ultimate goal of all attempts is to advance islet transplantation from glycemic control to a truly complete cure.

## Figures and Tables

**Table 1:** Selected treatment options for islet graft and islet transplant recipient on targets associated with IBMIR.

Donor (*in vitro* pretreatment)		Recipient (*in vivo* treatment)	
Agent	Mechanism	Agent	Mechanism
*Coagulation*			
Anti-TF mAb	Anti-TF	Anti-TF mAb	Anti-TF
Nicotinamide	Downregulation of TF expression on isolated islets	Heparin	Anticoagulation
Surface engineering of islets (i) Heparin coating (ii) PEG coating (iii) Composite islet-endothelial cell graft	Covering of islets to prevent direct exposure to blood	LMW-DS	Anticoagulation
		Melagatran	Anticoagulation via thrombin inhibition
		Nacystelyn	Anticoagulation, anti-inflammatory, & antioxidant effects
		Activated protein C	Anticoagulation, fribrinolysis, NF-*κ*B inhibition
		Thrombomodulin	Anticoagulation via thrombin inhibition
		Glycoprotein IIb/IIIa inhibitors	Inhibiting fibrinogen binding to the receptors to prevent platelet activation and aggregates

*Complement*			
*α*-Antitrypsin	Complement inhibition	LMW-DS	Complement inhibition
		Compstatin	Complement inhibition
		Cobra venom factor	Depleting the complements
		sCR1 (TP10)	Negative regulator of the complement cascade that inhibits both the classic and alternative pathways
		C5a inhibitory peptide (C5aIP)	Blocks the deleterious effects of C5a to reduce complement activation, the chemotactic effect, and inflammatory reactions

*Proinflammatory mediators*			
*α*-Antitrypsin	Blockade of PIC production	*α*-Antitrypsin	Blockade of PIC production
Antioxidant	Scavenging of reactive oxygen species produced the isolation process	15-DSG	Blockade of PIC production via NF-*κ*B inhibition
Statins	Protection against ischemia-reperfusion injury Inhibition of proapoptotic pathways	Anti-TNF-*α* mAb	Binding to TNF-*α* prevents the stimulation of its receptor
Heme oxygenase-1 induction	Anti-inflammatory effects via p38 MAPK-dependent pathway	IL-1 receptor antagonist	Prevention of IL-1*β* from binding to the IL-1 receptor
A-20 induction	Anti-inflammatory effects via NF-*κ*B inhibition potent antiapoptotic gene	Reparixin	Binding to CXCR1/2 to block the CXCL8/IL-8 axis
Active vitamin D	Anti-inflammatory effects by induction of protective gene expression	zVAD-FMK	Pan-caspase inhibitor that suppresses cell apoptosis
Withaferin A	Anti-inflammatory effects via NF-*κ*B inhibition	IDN-6556	Pan-caspase inhibitor that suppresses cell apoptosis
JNK inhibitor	Anti-inflammatory effects by reduction of PIC production Antiapoptotic effects via JNK inhibition	JNK inhibitor	Anti-inflammatory effects by reduction of PIC production Antiapoptotic effects via JNK inhibition
		GLP-1 R agonist (i) Exenatide (short half-life) (ii) Liraglutide (long half-life)	Reducing apoptosis due to oxidative stress & enhancing insulin release Anti-inflammatory & antioxidative properties

IBMIR = instant blood-mediated inflammatory reaction; PIC = proinflammatory cytokines; TF = tissue factor; LMW-DS = low-molecular-weight dextran sulfate; TNF-*α* = tumor necrosis factor-*α*; sCR1 = soluble complement receptor type 1; 15-DSG = 15-deoxyspergualin; GLP-1R = glucagon-like peptide-1 receptor; IL = interleukin; JNK = c-Jun N-terminal kinase; MAPK = mitogen-activated protein kinase; CXCL = CXC chemokine ligand; CXCR = CXC chemokine receptor.

**Table 2:** Selected immunosuppressive and anti-inflammatory agents used in islet transplantation.

Generic name	Trade name	Mechanism of action	Reference
*Induction (depletion of T cell or inhibition of T cell activation)*			
Antithymocyte globulin (ATG)	Thymoglobulin	Polyclonal antibody, profound T cell depletion	[100]
Muromonab-CD3	Orthoclone OKT3	Anti-CD3 mAb, T cell depletion	[101]
Alemtuzumab	Campath Lemtrada	Anti-CD52 mAb, T cell depletion	[102]
Basiliximab	Simulect	Anti-CD25 mAb IL-2 receptor antagonist	[103]
Daclizumab	Zenapax	Anti-CD25 mAb IL-2 receptor antagonist	[104]
*Maintenance (inhibition of T cell activation and proliferation)*			
Azathioprine	Imurel	Purine synthesis inhibitor Inhibition of T/B cell proliferation	[105]
Cyclosporine	Sandimmune Neoral	Calcineurin inhibitor Inhibition of T cell proliferation	[106]
Tacrolimus	Prograf Advagraf	Calcineurin inhibitor Inhibition of T cell proliferation	[94]
Mycophenolate mofetil (MMF)	CellCept	Purine synthesis inhibitor Inhibition of T/B cell proliferation	[107]
Sirolimus	Rapamune	mTOR inhibitor, inhibits T/B cell proliferation	[108]
Everolimus	Zortress/Certican	mTOR inhibitor	[106]
Etanercept	Enbrel	TNF-*α* inhibitor	[106]
Anti-CD154-mAb		Blockage of CD40/CD154 T cell costimulation	[109]
Abatacept (CTLA4-Ig)	Orencia (1st generation)	Blockage of B7/CD28 T cell costimulation	[110]
Belatacept (CTLA4-Ig)	Nulojix (2nd generation)	Blockage of B7/CD28 T cell costimulation	[111]
Leflunomide	Arava Lunava	Pyrimidine synthesis inhibitor, blockage of the proliferation of T/B cells	[112]
*Anti-inflammation*			
Adalimumab	Humira	Anti-TNF *α*-mAb (human)	[113]
Infliximab	Remicade	Anti-TNF *α*-mAb (chimeric human-mouse)	[114]
Etanercept	Enbrel	Soluble TNF receptor fusion protein	[106]
Anakinra	Kineret	IL-1*β* receptor antagonist	[115]
A1AT (alpha-1 antitrypsin)		Reduction in inflammatory cytokines	[54]
SP600125 (JNK inhibitor)		Inhibition of the production of PIC	[116]

PIC = proinflammatory cytokines; mAb = monoclonal antibody; TNF = tumor necrosis factor; IL = interleukin.

## Abbreviations

T1DM: Type 1 diabetes mellitus
IBMIR: Instant blood-mediated inflammatory reaction
IL-1*β*: Interleukin-1*β*
TNF-*α*: Tumor necrosis factor-*α*
TF: Tissue factor
VEGF: Vascular endothelial growth factor
MHC: Major histocompatibility complex
PRA: Panel-reactive antibody
DSA: Donor-specific antibody.

## Supplementary Materials

Supplementary Materials In the process of clinical islet allotransplantation, the transfused islet grafts through portal vein into the liver are subjected to multiple insults from the host, including the early massive islet loss due to hypoxia, instant blood-mediated inflammatory reactions, inflammatory cytokine injury, and late islet failure due to immune response, metabolic stress, and/or the chronic toxicity of the immunosuppressive drugs.
