# Supplementary figures and images for: The Fate of Allogeneic Pancreatic Islets following Intraportal Transplantation: Challenges and Solutions
Source: J Immunol Res. 2018 Sep 23;2018:2424586. doi: 10.1155/2018/2424586 (PMC6174795; doi:10.1155/2018/2424586)

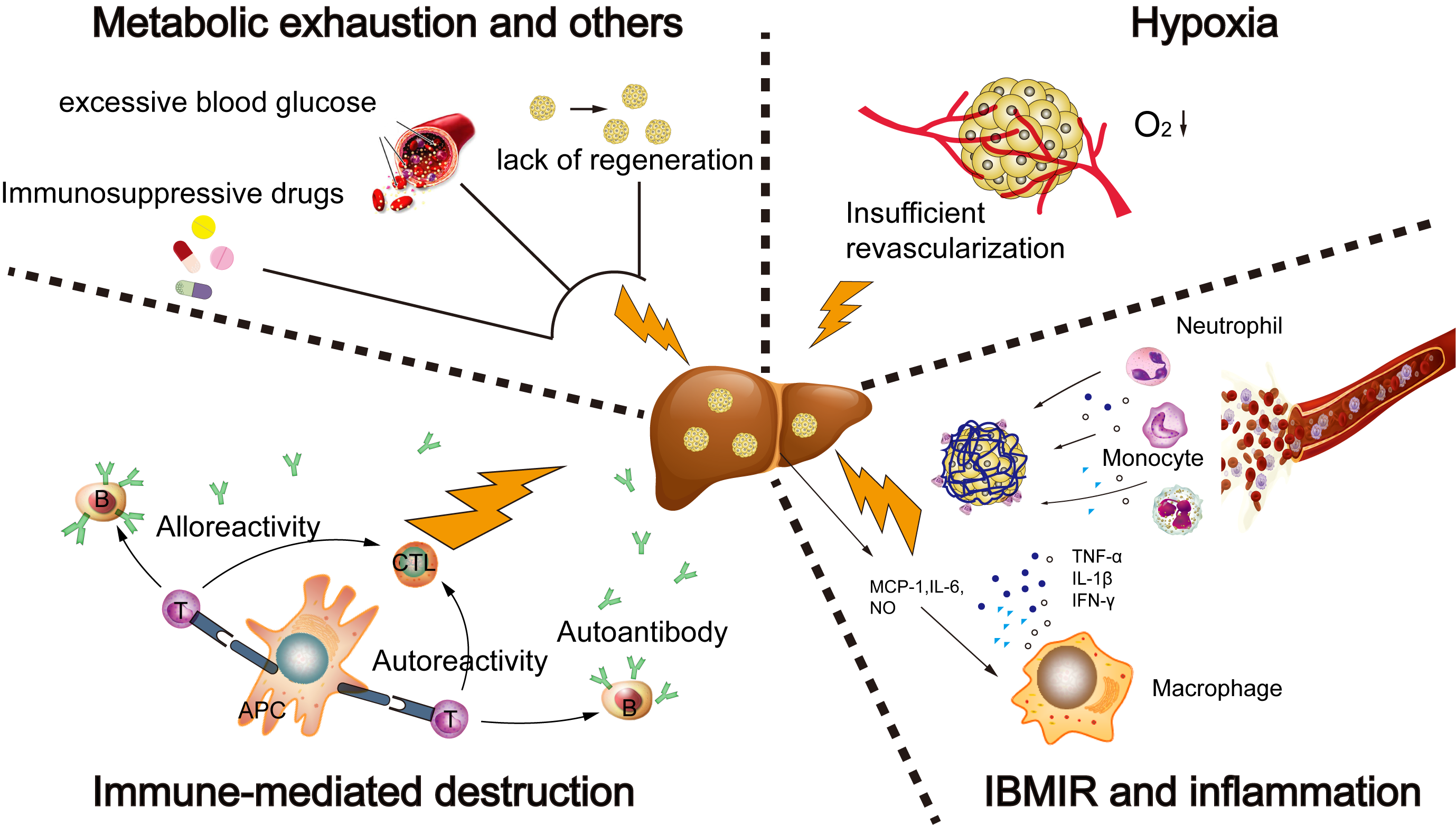

Supplement: Supplementary Materials — In the process of clinical islet allotransplantation, the transfused islet grafts through portal vein into the liver are subjected to multiple insults from the host, including the early massive islet loss due to hypoxia, instant blood-mediated inflammatory reactions, inflammatory cytokine injury, and late islet failure due to immune response, metabolic stress, and/or the chronic toxicity of the immunosuppressive drugs. [file 2424586.f1.tif]
